# O1.7. PROTEOMIC ANALYSIS OF BLOOD BASED SAMPLES FROM THE OPTiMiSE (OPTIMIZATION OF TREATMENT AND MANAGEMENT OF SCHIZOPHRENIA IN EUROPE) STUDY POINT TOWARDS COMPLEMENT PATHWAY PROTEIN CHANGES

**DOI:** 10.1093/schbul/sby015.189

**Published:** 2018-04-01

**Authors:** Melanie Föcking, Thomas Pollak, Patrick Dicker, Gerard Cagney, Inge Winter, Rene Kahn, Philip McGuire, David Cotter

**Affiliations:** 1 Royal College of Surgeons in Ireland; 2 Institute of Psychiatry, Psychology and Neuroscience, King’s College London; 3 Proteome Research Centre, University College Dublin; 4 University Medical Centre Utrecht; 8 Royal College of Surgeons in Ireland, Beaumont Hospital

## Abstract

**Background:**

The OPTiMiSE (Optimization of Treatment and Management of Schizophrenia in Europe) trial may help in the identification of predictors of treatment response. Medication naïve patients with first episode schizophrenia or schizophreniform disorder were enrolled in the study and treated open-label for a four-week period with amisulpride. PANSS ratings were undertaken at baseline and following the four-week treatment. 30 non-remitters (as defined by the Andreasen criteria) with the worst change in PANSS scores and the 30 remitters with the best change in PANSS scores were selected to represent good and poor outcome groups.

**Methods:**

We compared proteomic markers in serum collected prior to treatment in 30 patients who subsequently showed a good response to amisulpride (“responders”, and 30 patients who did not show a good response (“non-responders”). Serum samples were depleted using High Performance Liquid Chromatography (HPLC) attached to a MARS column to remove the 14 most abundant plasma proteins (albumin, IgG, antitrypsin, IgA, transferrin, haptoglobin, fibrinogen, alpha2-macroglobulin, alpha1-acid glycoprotein, IgM, apolipoprotein AI, apolipoprotein AII, complement C3, and transthyretin). The groups were matched for ethnicity, gender and age.

50 µg from each sample were reduced, alkylated, tryptically digested, then zip-tipped to concentrate and purify. Samples were then run for a 90-min gradient on a Thermo Scientific Q- Exactive Hybrid Quadrupole-Orbitrap Mass Spectrometer. Raw MS data were processed by MaxQuant software and searched against the human Uniprot database, for label free quantitation of peptides and proteins. False discovery rates (FDR) were set at 1% for both peptide and protein levels in target/decoy to minimize false positives. The match between runs feature was utilized.

**Results:**

Four hundred and sixty-four protein identifications were obtained. Samples were excluded where >30% of proteins were missing, and we used imputation, where the missingness depends upon a threshold of detection. This left 228 proteins for analysis. Of these, 21 were significantly different between responders and non-responders (p<0.05), one was FDR positive, one at trend FDR level. Pathway analysis (KEGG, David NIH) of the significant proteins determined “complement and coagulation cascades” to be the top pathway affected with six proteins from the list assigned to the pathway. These were CFI, C4A, C6, F9, VWF and SERPING1, all found to be up-regulated and with p-values ranging from 0.002 to 0.044. C6 is a constituent of the membrane attack complex (MAC) that plays a key role in the innate and adaptive immune response, while CFI belongs to the alternative pathway, C4A belongs to the classical pathway and F9 and VWF play roles in the intrinsic coagulation pathway.

**Discussion:**

These data complement results by Sekar et al., implicating excessive complement activity in the development of schizophrenia. Our data identifies the complement proteins in treatment response and this is also consistent with our previous findings of up-regulation of the complement pathway among those at risk of future psychotic experiences.

